# Effect of olfactory bulb pathology on olfactory function in normal aging

**DOI:** 10.1111/bpa.13075

**Published:** 2022-04-29

**Authors:** Cécilia Tremblay, Geidy E. Serrano, Anthony J. Intorcia, Lucia I. Sue, Jeffrey R. Wilson, Charles H. Adler, Holly A. Shill, Erika Driver‐Dunckley, Shyamal H. Mehta, Thomas G. Beach

**Affiliations:** ^1^ Departement of Neuropathology Banner Sun Health Research Institute Sun City Arizona USA; ^2^ Department of Economics Arizona State University Tempe Arizona USA; ^3^ Department of Neurology, Mayo Clinic College of Medicine Mayo Clinic Arizona Scottsdale Arizona USA; ^4^ Department of Neurology Barrow Neurological Institute Phoenix Arizona USA

**Keywords:** aging, Alzheimer's disease, amyloid β, Lewy body disease, olfactory bulb, tau, α‐synuclein

## Abstract

Decline of olfactory function is frequently observed in aging and is an early symptom of neurodegenerative diseases. As the olfactory bulb (OB) is one of the first regions involved by pathology and may represent an early disease stage, we specifically aimed to evaluate the contribution of OB pathology to olfactory decline in cognitively normal aged individuals without parkinsonism or dementia. This clinicopathological study correlates OB tau, amyloid β (Aβ) and α‐synuclein (αSyn) pathology densities and whole brain pathology load to olfactory identification function as measured with the University of Pennsylvania Smell Identification Test (UPSIT) and clinical data measured proximate to death in a large autopsy study including 138 cases considered non‐demented controls during life. Tau pathology was frequently observed in the OB (95% of cases), while both Aβ (27% of cases) and αSyn (20% of cases) OB pathologies were less commonly observed. A weak correlation was only observed between OB tau and olfactory performance, but when controlled for age, neither OB tau, Aβ or αSyn significantly predict olfactory performance. Moreover, whole brain tau and αSyn pathology loads predicted olfactory performance; however, only αSyn pathology loads survived age correction. In conclusion, OB tau pathology is frequently observed in normally aging individuals and increases with age but does not appear to independently contribute to age‐related olfactory impairment suggesting that further involvement of the brain seems necessary to contribute to age‐related olfactory decline.

## Graphical abstract

Findings of this clinicopathological correlative study suggest that olfactory bulb pathology is frequently observed in normally aging individuals and increases with age but does not appear to independently contribute to age‐related olfactory impairment.

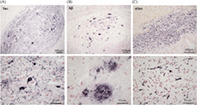

## INTRODUCTION

Decreased olfactory function is frequently observed in aging, and was suggested to be at least partly due to age‐related degenerative processes occurring in the brain. Moreover, olfactory dysfunction has repeatedly been reported to manifest early in neurodegenerative diseases and is expected to be a biomarker of the progression from normal cognition to mild cognitive impairment and dementia. Hence, it is important to investigate olfaction in normal aging with the aim to dissociate the olfactory impairment associated with neurodegenerative disease markers from that associated with the aging process alone.

Published clinicopathological correlative studies have established that older clinically normal subjects with incidental Lewy body disease (ILDB) or Alzheimer's disease (AD) lesions have lower scores on tests of olfactory function and that both Lewy and AD pathologies are present in olfactory‐relevant CNS regions including the olfactory bulb (OB). In fact, accumulation of both tau neurofibrillary (NF) and a‐synuclein (αSyn) pathology are frequent in the OB and were shown to appear early, while amyloid β (Aβ) plaques would appear in later stages and were less frequently detected in the OB [1, 2, 3, 4, 5, 6]. The presence of these pathologies in the OB was further shown to reflect the presence and severity of respective pathologies in other brain regions [1, 3, 6, 7].

These studies, however, did not have access to premortem olfactory performance of the subjects, and, to date, no studies have specifically correlated olfactory function with age and both Lewy body disease and AD pathology in the OBs of clinically normal elderly subjects. Hence, it is not clear to what extent the presence of pathology in the OB specifically contributes to olfactory impairment in normal aging subjects. Therefore, this study aimed to investigate the relationship between olfactory performance in normal aging and the presence of post‐mortem OB pathology.

## METHODS

Data from 138 cognitively normal controls subjects (65 men,73 women, mean age: 87.2 ± 6.9), without parkinsonism (mean Unified Parkinson's Disease Rating Scale [UPDRS] motor score: 7.8 ± 8.1) dementia or mild cognitive impairment as measured the Mini‐Mental State Exam (MMSE, mean score:28.2 ± 1.6), were available in the Banner Sun Health Research Institute Brain and Body Donation Program (BBDP) database [8]. All subjects had performed an olfactory test proximate to death (mean result 28.7 ± 6.3, interval test‐death: 28.7 ± 25.7 months) using the University of Pennsylvania Smell Identification Test (UPSIT). A full neuropathological examination was available including assignment of aSyn stage according to the Unified Staging System for Lewy Body Disorders (USSLBD), the AD Braak NF stage, the Thal amyloid phase for Aβ plaque brain distribution, the CERAD neuritic plaque score as well as a summary regional brain density measures for tau NF brain load and total plaques (for both, there is a total possible score of 15 based on summary of 0–3 scores in each of 5 regions: frontal, parietal, temporal, hippocampus CA1 and entorhinal/transentorhinal) and total brain load of aSyn pathology (total possible score of 40 based on summary of 0–4 scores for each of 10 brain regions) [8].

OB immunohistochemical (IHC) staining was performed for abnormally hyperphosphorylated tau protein (AT8 antibody), Aβ (6E10 antibody) as well as aSyn, pathology. OB pathology was semi‐quantitatively assessed, while blinded to clinical data, from 0–4 for tau and aSyn and from 0–3 for Aβ pathology.

## RESULTS

**FIGURE 1:**
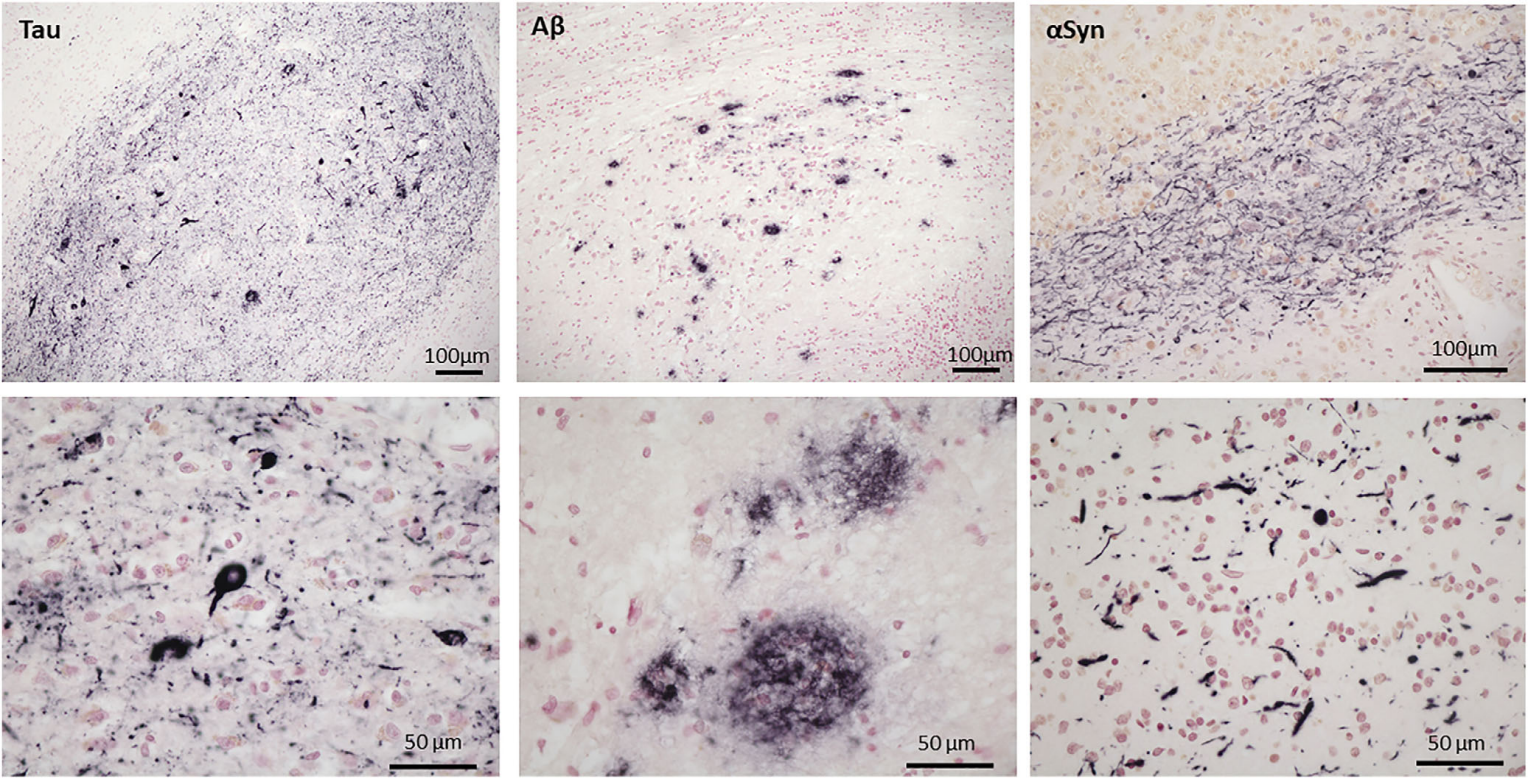
Photomicrographs depicting tau, Aβ and αSyn immunohistochemical pathologic inclusions in the OB at low and high magnifications

OB tau pathology was found in 125 out of 130 cases (95.2%) while the presence of OB Aβ pathology was found in in 35 out of 130 cases (26.9%) and OB αSyn pathology was found 28 out of 138 cases (20.3%) (Figure 1).

**FIGURE 2:**
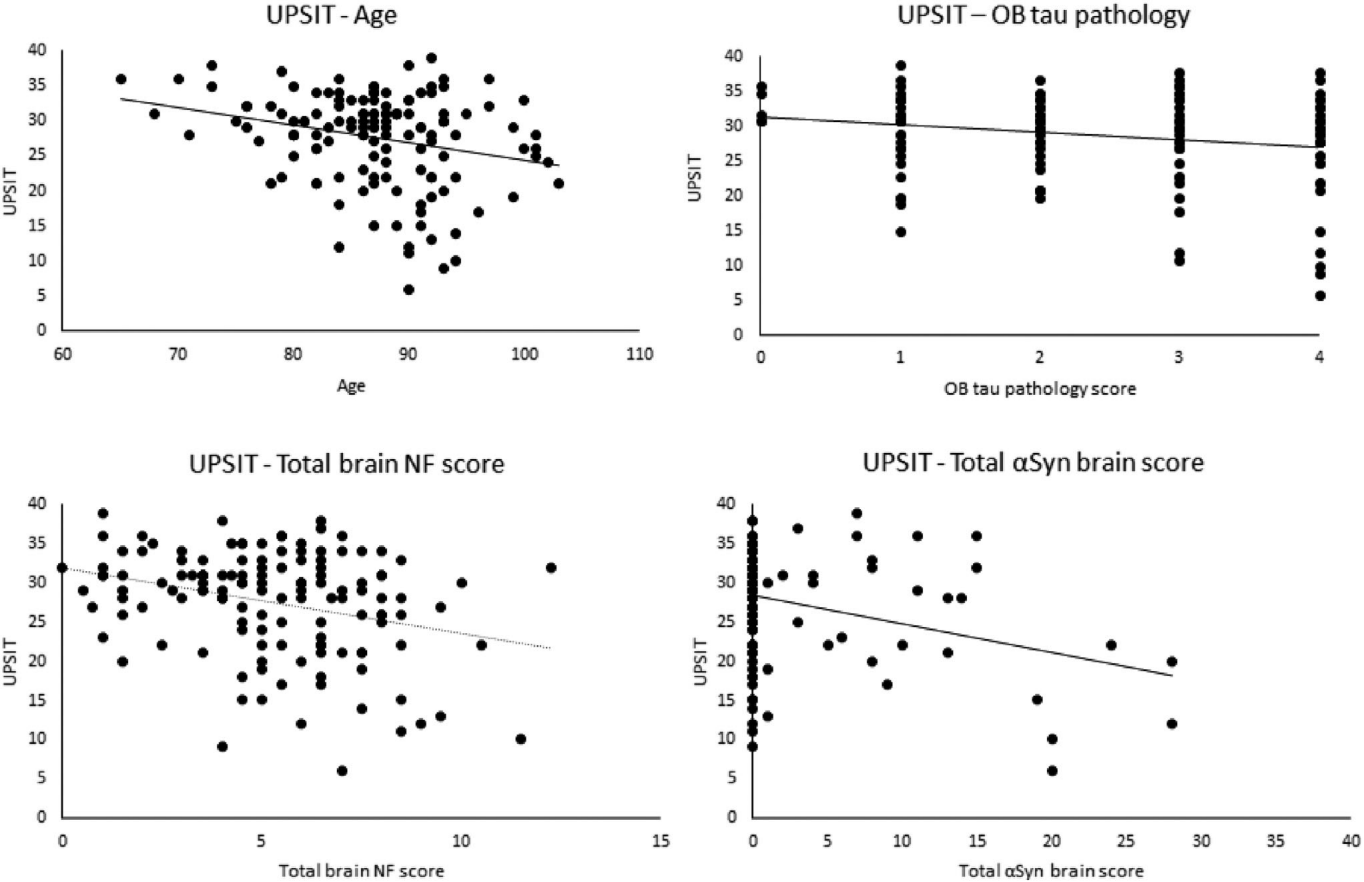
Significant correlations between UPSIT and age, OB tau pathology score, total NF and αSyn brain score

Significant Spearman correlations observed included OB tau with UPSIT score (Rho = −0.173, *p* = 0.025), age (Rho = 0.383, *p* < 0.0001), Braak NF stage (Rho = 0.483, *p* < 0.0001), MMSE score (Rho = −0.164, *p* = 0.033), UPDRS motor score (Rho = 0.163, *p* < 0.03), OB Aβ pathology score (Rho = 0.398, *p* < 0.0001), total brain NF score (Rho = 0.611, *p* < 0.0001), total brain plaque score (Rho = 0.427, *p* < 0.0001) and Thal amyloid phase (Rho = 0.406, *p* < 0.0001). UPSIT score correlated with age (Rho = −0.191, *p* = 0.01), Braak NF stage (Rho = −0.161, *p* = 0.03) and USSLBD stage (Rho = −0.158, *p* = 0.03) but did not correlate with OB Aβ pathology, OB αSyn pathology, MMSE, UPDRS motor score or Thal phase (Figure 2).

Multivariate linear regression analyses including all OB pathology scores (tau, Aβ, aSyn), whether without [*F*(3,129) = 1.404; *p* = 0.219; *R* ^2^ = 0.066] or with covariates for age, UPSIT‐ death interval, MMSE score and sex [F (6,126) = 1.404; *p* = 0.219; *R* ^2^ = 0.066], did not significantly predict UPSIT scores. When including only UPSIT score and OB tau pathology score, OB tau pathology score is a significant predictor of UPSIT score [*F*(1, 129) = 5.309; *p* = 0.023; *R* ^2^ = 0.040]. However, when controlling for age, even though the whole model is still significant [*F*(2, 129) = 3.609; *p* = 0.03; *R* ^2^ = 0.039], neither tau pathology score nor age is a significant independent predictor (both p > 0.05) of UPSIT score. When controlling for sex [*F*(2,129) = 2.636; *p* = 0.076; *R* ^2^ = 0.040], and adding UPSIT‐death interval [*F*(3,129) = 1.817; *p* = 0.147; *R* ^2^ = 0.041], MMSE score [*F*(4,126) = 1.035; *p* = 0.392; *R* ^2^ = 0.033], and age [F (5, 126) = 1.169; *p* = 0.328; *R* ^2^ = 0.046], none of the models significantly predicted UPSIT score.

Using whole brain pathology scores, both total brain NF score (Rho = −0.157, *p* = 0.03) total αSyn brain score (Rho = −0.269, *p* = 0.001), but not total brain plaque score (Rho = −0.132, *p* = 0.06), significantly correlated with UPSIT. A significant regression equation was found when controlling for age, sex, UPSIT‐ death interval and MMSE score, [*F*(4, 132) = 4.344; *p* = 0.003; *R* ^2^ = 0.120], with αSyn pathology brain score being the only independent predictor (*p* < 0.005) of UPSIT score.

## DISCUSSION

This is, to the best of our knowledge, the first study to assess the association between post‐mortem OB pathology and olfactory identification function measured before death in normal aging subjects without cognitive impairment or parkinsonism. We report frequent tau pathology in the OB while both Aβ and αSyn OB pathologies were less commonly observed. Even though a weak correlation was observed between OB tau and olfactory performance, OB tau pathology did not significantly predict olfactory function when controlled either for age and/or for sex and MMSE score. Furthermore, neither OB Aβ nor OB αSyn pathology were found to correlate with olfactory identification performance on the UPSIT. This suggest that OB pathology, even though increasing with age, does not seem to have an individual contribution to age‐related olfactory decline in cognitively normal individuals.

Our finding of frequent tau pathology in the OB of cognitively normal aged individuals is in agreement with other reports showing early tau pathology of the OB and other olfactory‐relevant regions in otherwise healthy individuals [2, 3, 5, 9], OB tau pathology being even more frequent that previously suggested and present as early as in Braak NF stage 1 [7]. We further report a correlation between age and OB tau pathology score which is concordant with studies showing increases in OB tau pathology as a function of age in non‐demented individuals [6]. Overall, this result indicates that olfactory impairment and OB tau pathology both increase with age but are so closely correlated that their individual contributions are inseparable. Our results suggest that tau pathology in the OB can be histologically severe in cognitively normal controls but may not directly affect neuronal function and thus olfactory processing. The relatively minor impact of OB pathology on olfactory performance in these normally aging subjects may have larger clinical implications as this suggests that these common age‐related neurodegenerative processes have early stages when neuronal function remains relatively spared. Molecular therapies capable of slowing or halting these processes may therefore be able to prevent the ultimate losses not only of olfactory function but also of cognitive impairment and movement abnormalities.

It is known that the sense of olfaction involves not only the olfactory bulb but also its closely‐connected brain regions including the piriform cortex, amygdala, entorhinal area, and orbitofrontal cortex, as well as other brain regions that contribute to general cognitive ability. This hypothesis is supported by the much stronger associations of whole brain pathology scores with UPSIT, despite the clinically unimpaired status of these subjects. However, again the association with tau pathology is negated when the additional variables including age or MMSE score are included in the models, while the significant association of UPSIT with αSyn pathology total brain score is preserved, suggesting that αSyn pathology has a stronger relationship with olfactory performance than does tau pathology, as previously reported [10].

In conclusion, OB tau pathology is frequently observed in normally aging individuals and increases with age but does not appear to independently contribute to age‐related olfactory impairment. Future studies should further investigate OB pathology together with a set of other olfactory‐relevant brain regions to better assess the link between olfactory function and neurodegenerative pathology.

## CONFLICT OF INTEREST

The authors declare no conflict of interest.

## AUTHOR CONTRIBUTIONS

Conception and design: CT, GES, AJI, LIS, JRW, CHA, HAS, ED, SM, TGB; Experimentations: CT, GES, AJI, LIS, JRW, CHA, HAS, ED, SM, TGB; Writing the first draft of the manuscript: CT, GES, TGB; Corrections and approbation of final manuscript: CT, GES, AJI, LIS, JRW, CHA, HAS, ED, SM, TGB.

## Data Availability

Data will be made available upon request to the corresponding author.
